# Characterization of aortic aging using 3D multi-parametric MRI-long-term follow-up in a population study

**DOI:** 10.1038/s41598-023-33219-7

**Published:** 2023-04-18

**Authors:** Sophie Loose, Demetris Solou, Christoph Strecker, Anja Hennemuth, Markus Hüllebrand, Sebastian Grundmann, Alexander Asmussen, Martin Treppner, Horst Urbach, Andreas Harloff

**Affiliations:** 1 Department of Neurology and Neurophysiology, University Medical Center Freiburg, University of Freiburg, Breisacher Straße 64, 79106 Freiburg, Germany; 2 Faculty of Medicine, University of Freiburg, Freiburg, Germany; 3 Fraunhofer MEVIS, Bremen, Germany; 4 Institute of Computer-Assisted Cardiovascular Medicine, Charité-Universitätsmedizin Berlin, Berlin, Germany; 5 Department of Cardiology and Angiology I, Heart Center Freiburg University, University of Freiburg, Freiburg, Germany; 6 Institute of Medical Biometry and Statistics, Faculty of Medicine, Medical Center, University of Freiburg, Freiburg, Germany; 7 Department of Neuroradiology, Medical Center, University of Freiburg, Freiburg, Germany

**Keywords:** Cardiovascular biology, Cardiovascular diseases, Atherosclerosis, Blood flow, Biomarkers

## Abstract

We comprehensively studied morphological and functional aortic aging in a population study using modern three-dimensional MR imaging to allow future comparison in patients with diseases of the aortic valve or aorta. We followed 80 of 126 subjects of a population study (20 to 80 years of age at baseline) using the identical methodology 6.0 ± 0.5 years later. All underwent 3 T MRI of the thoracic aorta including 3D T1 weighted MRI (spatial resolution 1 mm^3^) for measuring aortic diameter and plaque thickness and 4D flow MRI (spatial/temporal resolution = 2 mm^3^/20 ms) for calculating global and regional aortic pulse wave velocity (PWV) and helicity of aortic blood flow. Mean diameter of the ascending aorta (AAo) decreased and plaque thickness increased significantly in the aortic arch (AA) and descending aorta (DAo) in females. PWV of the thoracic aorta increased (6.4 ± 1.5 to 7.0 ± 1.7 m/s and 6.8 ± 1.5 to 7.3 ± 1.8 m/s in females and males, respectively) over time. Local normalized helicity volumes (LNHV) decreased significantly in the AAo and AA (0.33 to 0.31 and 0.34 to 0.32 in females and 0.34 to 0.32 and 0.32 to 0.28 in males). By contrast, helicity increased significantly in the DAo in both genders (0.28 to 0.29 and 0.29 to 0.30, respectively). 3D MRI was able to characterize changes in aortic diameter, plaque thickness, PWV and helicity during six years in our population. Aortic aging determined by 3D multi-parametric MRI is now available for future comparisons in patients with diseases of the aortic valve or aorta.

## Introduction

Progressive atherosclerosis of the aorta leads to an increase of aortic diameter, wall thickness and stiffness^1^. Aortic pulse wave velocity (PWV) reflects stiffness and is a strong predictor of future cardiovascular events and all-cause mortality^2^. Measuring PWV offers the chance to monitor vascular ageing, identify subjects at risk for cardiovascular events and intervene timely through lifestyle modifications and medication^3^. Consequently, PWV has been recommended as an independent parameter for individual risk assessment^4,5^. Helicity is another promising hemodynamic parameter representing the rotational component of blood flow. Physiological helicity seems to be atheroprotective while abnormal helicity has been associated with aortic dilation and aneurysms. Therefore, quantification of helicity is of high interest especially in patients with diseases of the aortic valve or the aorta^6–8^.

Growth of the diameter of the ascending and descending aorta was studied using CT-angiography in ca. 2000 subjects between 50 and 70 years of age of the Danish population. Annual progression was 0.1 mm per year and comparable to other population studies (growth rates = 0.1–0.2 mm/year) while an increase of 0.5 mm/year was considered the upper limit of normal^9^. Aortic wall thickness increased by 0.03 mm/year in 423 subjects of a population study using two-dimensional (2D) MRI in the descending aorta and during 10-year-follow-up^10^. PWV was a strong and independent predictor of cardiovascular events and all-cause mortality in a large meta-analysis including 16,000 patients examined by Doppler flow or tonometry and followed over 8 years^11^. However, PWV can also be measured using 2D phase-contrast MRI offering the possibility to exclusively study stiffness of the thoracic aorta. This method was applied in 1160 participants of the multi-ethnic study of atherosclerosis (MESA) in subjects aged 45–84 years. PWV increased by a median of 18% in 10 years and was associated with baseline age and the presence of hypertension^12^.

Four-dimensional (4D) flow MRI is a newer technology and was used for the assessment of aortic PWV in healthy volunteers, acute stroke patients and in a population study demonstrating high accuracy even in patients with complex aortic geometries^13–16^. 4D flow MRI offers the unique opportunity to combine measurement of PWV of the thoracic aorta with the measurement of aortic diameter and wall thickness, allowing multi-parametric assessment of individual atherosclerosis^16^. In addition, 4D flow MRI allows visualization and measurement of three-dimensional (3D) helicity of aortic blood in vivo and to discriminate physiological from pathological blood flow pattern^6–8,17^. Helical flow obviously induces relative uniformity of wall shear stress, inhibits flow stagnation and separation and prevents accumulation of LDL-cholesterol at the vessel wall. Thus, it seems to be important for blood transportation in arteries and protection from atherosclerosis^6^.

To date, there is no longitudinal study characterizing age-specific aortic aging in a population study using a thorough 3D MRI-based analysis of both morphological and hemodynamic parameters. Data of the progression of aortic aging in a population study would be highly valuable as reference values and for a comparison with patients with diseases of the aortic valve or aorta. It would allow to determine higher-than-average aging in patients and to guide individual treatment. For these reasons, we performed comprehensive 3D MRI follow-up examinations in a population^16^ six years after baseline in order to characterize both morphological and functional aortic aging.

## Methods

### Study population

Between October 2012 and August 2014 we performed a cross-sectional observational study of the population of our city based on data obtained from the local residents’ registration office. 3500 age-stratified and randomly selected residents were contacted by mail and asked to participate. 308 subjects responded, and were contacted consecutively by phone and recruited on a first-come, first-served basis. Due to low recruitment in 20–40-year-old men, we also advertised this study in our hospital. Finally, 126 subjects (ca. 10 females and ca. 10 males per decade) between 20 and 80 years of age were consecutively and prospectively included (for details see^16^). The following subjects were included: citizens of the city of Freiburg between 20–80 years of age. Contraindications against MRI (such as claustrophobia, pacemaker, pregnancy etc.) or unwillingness or inability to participate in the study and/or to give written informed consent were exclusion criteria of our study.

From May 2019 to February 2020, we performed the follow-up study. We contacted all participants again by mail and phone and asked them to undergo repeated medical interview on site, MRI examination and transthoracic echocardiography (TTE). Part of the study population was lost to follow-up for the following reasons: refusal of participation (n = 16), contraindications against MRI such as pregnancy, breastfeeding, new pacemaker, or cardiac arrhythmia disturbing the ECG-trigger in MRI (n = 6). 14 patients did not reply to repeated mail and phone contacts (n = 14), two patients died and another four could not be measured since we stopped follow-up measurements due to the COVID-19 pandemic at the end of February 2020. In addition, artifacts in MR images probably caused by incorrect PEAK (parallel MRI with Extended and Averaged GRAPPA Kernels) GRAPPA (GeneRalized Autocalibrating Partial Parallel Acquisition) acquisition or external radiofrequency sources disturbed image acquisition (n = 2). Incomplete 4D flow measurement due to irregular heart rate and respiration (n = 2) and a problem with the electrocardiogram (ECG)-trigger in MRI (n = 1) made data analysis impossible in these five patients. As a result, we were able to evaluate complete datasets of 80 subjects (63.5% of the subjects at baseline) at follow-up six years later.

The ethics committee of the Albert Ludwig University of Freiburg, Germany, approved the baseline and follow-up study. In addition, we obtained written informed consent from all participants. All experiments were performed in accordance with relevant guidelines and regulations.

### Baseline characteristics

Cardiovascular risk factors and demographics of the 80 participants at the time of follow-up were determined by interview on site. We measured blood pressure at the left upper arm in a supine position after 5 min. rest before and after MRI examination and every 15 min. during MRI and documented heart rate every 3 min. during 4D flow MRI lasting 15–30 min. depending on individual breathing and heart rate.

### Transthoracic echocardiography

We performed transthoracic echocardiography (TTE) to detect relevant aortic valve stenosis, aortic valve insufficiency and reduced left ventricular systolic function, which would have an impact on aortic blood flow, especially on pulse wave velocity and helicity. TTE was performed both at baseline and follow-up to detect possible changes of cardiac parameters over time.

All participants underwent transthoracic echocardiography (Affiniti ultrasound system, 2 MHz transducer; Philips, Leiden, Netherlands) based on the recommendations and standards of the American Society of Echocardiography^18,19^. The TTE protocol was comparable to that at baseline but the ultrasound machine and physicians from the Department of Cardiology performing ultrasound were different. Two physicians from the Department of Cardiology performed standard TTE under supervision by a cardiologist.

TTE was performed on the same day as MRI except in one patient who received TTE the day after MRI examination. All standard 2D transthoracic echocardiographic images, standard M-mode and Doppler images were obtained in apical, parasternal long and short axis view and in subcostal view. Left ventricular (LV) systolic and diastolic function, LV ejection fraction (EF), right ventricular systolic function, wall-motion and valvular function and morphology were assessed. Left atrial volume, left and right ventricular dimensions, left ventricular wall thickness and diameters of the ascending aorta were calculated. Flow velocity measurements were performed through the tricuspid, pulmonary outflow, mitral, and aortic outflow regions.

### MRI measurements

MRI examinations were conducted on a routine 3 Tesla MRI (TIM Trio, Siemens, Erlangen, Germany) using a standard 12-element body coil. The MRI protocol used for follow-up was identical to that used at baseline^16^.

#### Measurement of aortic diameter and plaque thickness

T1 weighted bright-blood MRI (3D gradient echo sequence, echo time/repetition time (TE/TR) = 1.89/152.53 ms, flip-angle = 20°, acceleration = GRAPPA (*R* = 2, 32 ref. lines) with a spatial resolution of 1.1 × 0.9 × 1.1mm^3^ allowed off-line measurements of the maximum diameter of the ascending (AAo) and descending aorta (DAo). We performed these measurements at the level of the pulmonary artery and of the aortic arch between the outlet of the brachiocephalic artery (BCA) and the left common carotid artery (CCA). Maximum diameter and plaque thickness were determined manually in each of these three aortic segments using electronic calipers and a routine picture archiving and communication system (PACS) (IMPAX EE, Agfa HealthCare, Bonn, Germany). Values were determined in consensus reading by two neurologists with an experience of each > 7 years in MRI research including imaging of aortic atherosclerosis. ECG-trigger and navigator gating minimized motion artifacts^19^. We dichotomized circumscribed wall thickening as atheroma < 4 mm and ≥ 4 mm. The latter were defined as complex plaques are known to be associated with an increased risk of stroke^20^.

#### Measurement of aortic blood flow

4D flow MRI acquired time-resolved 3D blood flow information of the thoracic aorta. All experiments used prospective ECG- and navigator-gating to allow free breathing^21^. Parameters were TE/TR = 2.54/5 ms, flip angle = 7°, temporal resolution = 20 ms, matrix size = 340 × 255 × 75, bandwidth = 450 Hz/pixel, spatial resolution = 2.5 × 2.1 ×  2.5mm^3^, velocity sensitivity along all three directions = 150 cm/s, and parallel imaging (PEAK-GRAPPA) along the phase encoding direction (y) with an acceleration factor of R = 5 (20 reference lines).

**Figure 1:**
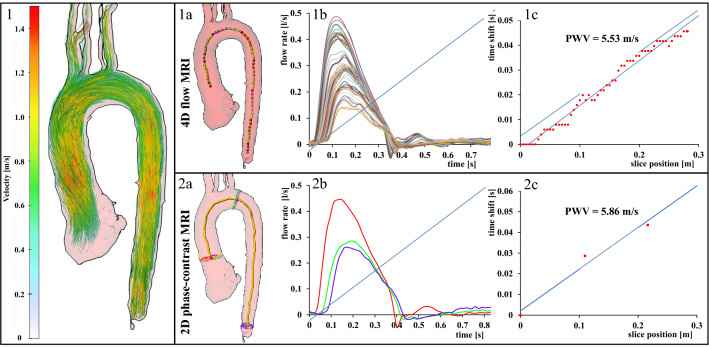
Pulse wave velocity calculations based on 4D flow MRI and 2D phase-contrast MRI. 4D flow MRI (upper row): 3D blood flow velocities are visualized using color-coded streamlines representing true blood flow velocities (1). Analysis planes were automatically placed at a distance of 5 mm along the automatically generated centerline (**1a**, shown here as colorful intersection points). Blood flow volume at each of these analysis planes is displayed over the cardiac cycle (**1b**). We calculated PWV from flow curves and a linear slope fitted to all single values is drawn (**1c**). 2D phase-contrast MRI (lower row): we added three single analysis planes to the centerline generated based on 4D flow data (**2a**). Blood flow volume is displayed over the cardiac cycle at each of these three planes (**2b**). PWV is calculated based on this information and a linear slope is drawn (**2c**).

2D phase-contrast MRI with higher resolution (spatial/temporal resolution = 1.3mm^2^/10.6 ms) was performed for comparison with 4D flow MRI at three predefined positions and perpendicular to the aorta: (a) in the ascending aorta above the aortic bulb, (b) in the aortic arch behind the outlet of the left subclavian artery and (c) in the descending aorta 1–2 cm above the summit of the liver in a coronal orientation (see Fig. 1). Further parameters were TE/TR = 10.6/2.9 ms, flip angle = 7°, matrix size = 140 × 256, bandwidth = 450 Hz/pixel, velocity sensitivity through plane = 150 cm/s, and parallel imaging (GRAPPA) with an acceleration factor of R = 5 (20 reference lines). Measurements were ECG-triggered and navigator gating allowed free breathing.

### Calculation of global pulse wave velocity based on 4D flow MRI

Datasets of 4D flow MRI measurements were analyzed off-line using MEVISFlow software (Fraunhofer MEVIS, Bremen, Germany)^22,23^. After corrections for eddy-currents and phase-wraps, we segmented the aorta semi-automatically. A centerline was automatically positioned along the entire thoracic aorta starting from the aortic root to the level of the diaphragm. Then, MRI analysis planes were automatically distributed along the centerline after manually setting start and endpoints within the aortic lumen. They were orientated normal to the aorta with an inter-plane distance of 5 mm. This resulted in 58 ± 7 analysis planes in our study participants depending on the individual length of the thoracic aorta (28.2 ± 3.7 cm) (see Fig. 1). The software defined a contour surrounding the lumen automatically based on the 3D segmentation of the aorta^22^. These steps were performed by two observers and took approximately 10–20 min. per subject. Calculation of the study parameters (global and regional PWV, segmental helicity) was performed automatically and in a few minutes by the software.

Individual pulse wave velocity in m/s was calculated based on these planes using the time-to-foot (TTF), 50%-rule (time point where the flow rate is half of the peak flow rate), and cross-correlation (XCor) as described previously^14,22,23^. For all statistical analyses, we used the calculation of PWV by XCor because this was the most stable parameter in the analysis of baseline data^13^. Using all three algorithms, the automated computing of PWV took 20–40 s when using a 2.6 GHz Intel Core i7 computer or a 3.4 GHz Intel Core i7 computer with 32 Gb RAM working memory.

### Calculation of regional pulse wave velocity based on 4D flow MRI

4D flow MRI included information of time-resolved 3D blood flow of the entire thoracic aorta and thus allowed calculation of regional PWV by intersecting the aorta: we considered all analysis planes along the centerline from the starting point in the proximal ascending aorta to the distal wall of the outlet of the left subclavian artery (LSA) to measure PWV of the AAo + AA. Regional PWV of the DAo was calculated by incorporating all analysis planes from the level of the LSA outlet reaching to the diaphragm. Regional PWV was then calculated as described for global aortic PWV (see above).

### Calculation of global pulse wave velocities based on 2D phase-contrast MRI

In contrast to the baseline examinations^16^ we calculated PWV by importing three analysis planes of 2D phase-contrast MRI in the ascending and descending aorta into the MEVISFlow software (see above) for comparison with 4D flow MRI. This approach allowed a better evaluation of measurement accuracy of PWV obtained by 4D flow MRI.

We measured the distance between these three planes using the centerline of the 4D flow MRI analysis. The position of the first (ascending aorta) and third plane (distal descending aorta above the diaphragm) defined the start and end points of the centerline. In each plane, blood-volume curves were extracted, and PWV in m/s was calculated using the methods mentioned above (see Fig. 1). Four 2D phase-contrast datasets were spoiled due to a failure of the ECG-trigger, one measurement showed phase-wrapping artifacts, and in another subject, the first plane was positioned too proximal cutting the aortic valve. Thus, we compared 2D phase-contrast datasets of 74 study participants with 80 datasets obtained from 4D flow MRI.

### Calculation of segmental helicity of aortic blood flow

**Figure 2:**
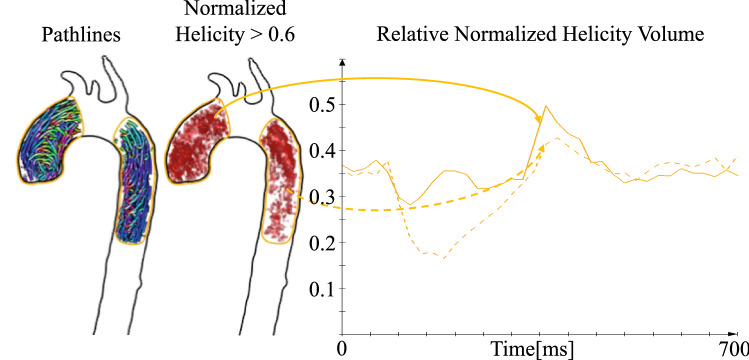
Time-resolved three-dimensional helicity in the thoracic aorta. Concept for the calculation of the relative thresholded normalized helicity for ascending (Asc) and descending aorta (Desc). The pathlines in the left part of this figure indicate helical flow patterns in the diastolic phase. The middle image shows the thresholded normalized helicity calculated for the corresponding timeframe. The diagram on the right demonstrates the resulting relative volumes ${\rm{VH}}_{{\rm{s}},{\rm{t}}}$ for the ascending and descending aorta.

The relative portion of normalized helical flow per time point and vessel segment was calculated as suggested by Garcia et al.^24^ and is illustrated in Fig. 2. Normalized helical flow ${{\varvec{L}}{\varvec{N}}{\varvec{H}}}_{{\varvec{x}},{\varvec{y}},{\varvec{z}},{\varvec{t}}}$ per image voxel ${v}_{x,y,z,t}$ was computed based on the on the velocities ${{\varvec{V}}}_{{\varvec{x}},{\varvec{y}},{\varvec{z}},{\varvec{t}}}$ and the corresponding vorticity vectors ${{\varvec{\omega}}}_{{\varvec{x}},{\varvec{y}},{\varvec{z}},{\varvec{t}}}=curl({{\varvec{V}}}_{{\varvec{x}},{\varvec{y}},{\varvec{z}},{\varvec{t}}})$: ${{\varvec{L}}{\varvec{N}}{\varvec{H}}}_{{\varvec{x}},{\varvec{y}},{\varvec{z}},{\varvec{t}}}=\boldsymbol{ }\frac{{{\varvec{V}}}_{{\varvec{x}},{\varvec{y}},{\varvec{z}},{\varvec{t}}}\cdot{{\varvec{\omega}}}_{{\varvec{x}},{\varvec{y}},{\varvec{z}},{\varvec{t}}}}{\left|{{\varvec{V}}}_{{\varvec{x}},{\varvec{y}},{\varvec{z}},{\varvec{t}}}\right|\left|{{\varvec{\omega}}}_{{\varvec{x}},{\varvec{y}},{\varvec{z}},{\varvec{t}}}\right|}$**.** A high absolute value indicates increased helicity. The quantitative assessment per vessel segment ${\varvec{s}}$ was implemented via thresholding with $th=0.6$ as suggested by Garcia et al.^24^. In addition to the calculation of the absolute volumes of voxels with ${{\varvec{L}}{\varvec{N}}{\varvec{H}}}_{{\varvec{x}},{\varvec{y}},{\varvec{z}},{\varvec{t}}}>0.6$, we calculated the volume portions through normalization with the segment volume ${v}_{s,t}=\left|{v}_{x,y,z,t}\left|{v}_{x,y,z,t}\in s\right.\right|$: ${VH}_{s,t}=\frac{\left|\left\{{v}_{x,y,z,t}|{v}_{x,y,z,t}\in {\varvec{s}}\wedge {\boldsymbol{ }{\varvec{L}}{\varvec{N}}{\varvec{H}}}_{{\varvec{x}},{\varvec{y}},{\varvec{z}},{\varvec{t}}}>th\right\} \right|}{{v}_{s,t}}$ (see supplemental Tables 1 and 2). By using volume portions per vessel segment the influence of individual vessel segment size is reduced.

### Measurement accuracy of PWV based on 4D flow MRI data

18 healthy volunteers were recruited from the staff of our hospital by advertising this study by notice and in the intranet at the time of follow-up measurements. We recruited two females and two males per decade between 20 and 60 and one female and one male between 60 and 65 years of age. All volunteers underwent the MRI protocol described above at the first appointment and a second time 40.3 ± 12.7 days later for the test of reproducibility.

PWV was calculated based on 4D flow MRI as described above. Observer 1 performed analysis of the first MRI at one time point and again at least four weeks later and was blinded to the first results (= intra-observer agreement). Observer 2 was carefully instructed to the fashion of data analysis and calculated PWV of the first MRI measurement blinded to the results of observer 1 (= inter-observer agreement). Finally, observer 1 analyzed PWV based on the second MRI examination blinded to the results of the first MRI to evaluate scan-rescan reproducibility.

### Statistical analysis

Data are presented as mean (± standard deviations) or median (interquartile range) for continuous variables and absolute frequencies and percentages for categorical variables. We analyzed differences between the two time points of aortic diameter and plaque thickness, global and regional PWV and helicity using a Wilcoxon signed-rank test. We further analyzed the difference between men versus women and changes in patients’ characteristics and TTE parameters between baseline and follow-up examination in a two-sample t-test. All tests were two-sided with 0.05 as the statistical significance level.

Linear regression analyses were performed to predict changes in PWV, aortic diameter, aortic plaque thickness and local normalized helicity volumes (follow-up—baseline), where we adjusted for age and sex in all models. Specifically, in the linear model for PWV, we predicted change in baseline to follow-up by arterial hypertension (binary), mean of aortic plaques (across AAo, AA, and DAo), and mean of aortic diameters (across AAo, AA, and DAo). To predict changes in aortic diameter, aortic plaque thickness and helicity, we used arterial hypertension (binary) and PWV at baseline as potential predictors. We fitted a separate model for each region (AAo, AA, and DAo). For all models, we performed a complete case analysis. For internal model validation, we used R^2^ (coefficient of variation) and examined the residuals.

Intra- and inter-observer agreement and scan-rescan reproducibility of PWV calculation, based on 4D flow MRI and 2D phase-contrast MRI data, as well as the comparison of PWV determined by the two methods are reported by mean difference and limits of agreement using a Bland–Altman plot. In addition, the intraclass correlation coefficient (ICC) for the scan-rescan reliability and for the comparison of PWV determined by 4D flow and 2D phase-contrast MRI was calculated using the following quality of agreement: less than 0.40—poor, between 0.40 and 0.59—fair, between 0.60 and 0.74—good, and between 0.75 and 1.00—excellent.

Statistical analyses were performed using the R programming language^25^. All analyses were exploratory in nature. As a result, p-values and 95% confidence intervals were not corrected for multiple comparisons, and inferences drawn from them may not be reproducible.

## Results

### Study cohort

Mean time interval between baseline and follow-up measurements in our population was 6.0 ± 0.5 years. Table 1 demonstrates cardiovascular risk factors of our cohort at baseline and follow-up. All participants were Caucasians and mean age was 53.9 ± 14.6 years at baseline and 59.9 ± 14.6 years at follow-up. Mean age of the 18 healthy volunteers recruited for determination of measurement accuracy of PWV calculations was 42.7 ± 13.3 years.

**Table 1:** Patients’ characteristics and cardiovascular risk factors of study participants.

Characteristics	Baseline (N = 80)	Follow-up (N = 80)	p-value
Age, years (± SD)	53.9 (14.6)	59.9 (± 14.6)	< 0.001
Female, n (%)	42 (52.5)	42 (52.5)	1.000
Hypertension, n (%)	12 (15)	19 (23.8)	0.052
Hypercholesterolemia, n (%)	15 (18.8)	17 (21.3)	0.640
Diabetes, n (%)	1 (1.3)	3 (3.8)	0.159
Smoker, n (%)	12 (15)	6 (7.5)	0.083
BMI, kg/m^2^ (± SD)	24.8 (3.8)	24.9 (4.5)	0.352
Prior stroke, n (%)	1 (1.3)	1 (1.3)	1.000
Coronary heart disease, n (%)	0 (0)	3 (3.8)	0.083
Peripheral arterial disease, n (%)	0 (0)	1 (1.3)	0.320
Mean systolic BP, mmHg (± SD)	127.8 (17.1)	123.2 (16.9)	0.009
Mean diastolic BP, mmHg (± SD)	80.1 (8.3)	74.8 (12.2)	< 0.001
Heart rate, bpm (± SD)	67.4 (10.5)	67.0 (9.5)	0.728

*BMI* body mass index, *SD* standard deviation, *BP* blood pressure, *bpm* heart rate.

Of the baseline study cohort (= 126 subjects), 80 subjects participated in follow-up (each 18–19 subjects of the initial 50–59 and 60–69 decades, 13–14 subjects of the initial 40–49 and 70–80 decades and 6–10 subjects of the initial 20–29 and 30–39 decades). So especially younger participants were lost to follow-up. Among the 80 subjects, the incidence of hypertension, diabetes, hypercholesterolemia and coronary heart diseases increased, the level of BMI remained the same and the incidence of smoking decreased during time. Blood pressure measured during the study was lower at follow-up than at baseline, whereas heart rate was the same. None of the changes were significant except for blood pressure. Table 2 demonstrates the results of transthoracic echocardiography (TTE). TTE values were normal in all but two individuals showing a reduced left ventricular function (ejection fraction = 50%). None of the patients had a bicuspid aortic valve and there were no significant changes in left ventricular ejection fraction and aortic valve disease over time. Maximum velocity across the aortic valve increased significantly but none of the patients had a severe aortic valve stenosis.

**Table 2:** Echocardiographic variables of the study participants.

Characteristics	Baseline	Follow-up	P value
Left ventricular ejection fraction, % (± SD)	55.2 (± 1.3)	55.1 (± 1.6)	0.114
Aortic valve insufficiency			0.418
Grade 0°, n (%)	74 (93.7)	76 (96.2)	
Grade I°, n (%)	4 (5.1)	3 (3.8)	
Grade II°, n (%)	1 (1.3)	0 (0)	
Aortic valve stenosis			0.320
Mild, n (%)	0 (0)	11111(0)	
Moderate, n (%)	0 (0)	1 (1.3)	
Maximum velocity across aortic valve—m/s (± SD)	1.22 (± 0.21)	1.34 (± 0.32)	< 0.001

Please note that TTE information was available in 79 patients of follow-up. Baseline values are provided for these 79 patients. At follow-up, maximum velocity across aortic valve were available in 78 of 80 patients. We detected no bicuspid aortic valve in our patients. N = number of subjects, SD = standard deviation.

### Progression of aortic diameter, plaque thickness, pulse wave velocity and helicity

Table 3 demonstrates the progression and regression of morphological and hemodynamic study parameters during 6 years. Mean diameter of the ascending aorta decreased and plaque thickness increased significantly in the aortic arch and descending aorta only in women.

**Table 3:** Aortic parameters in the 80 participants at baseline and follow-up.

Parameter	Segment	Females			Males		
		Baseline, N = 42^a^	Follow-up, N = 42^a^	p-value^b^	Baseline, N = 38^a^	Follow-up, N = 38^a^	p-value^b^
Diameter (mm)	Aao	32.3 (3.9)	31.6 (3.6)	0.032	34.4 (4.8)	34.3 (4.1)	0.669
	AA	27.6 (3.5)	27.7 (3.2)	0.653	30.1 (4.3)	29.7 (3.2)	0.072
	Dao	25.1 (3.4)	25.0 (3.2)	0.704	27.3 (4.5)	27.4 (3.6)	0.819
Plaques (mm)	Aao	0.84 (1.31)	0.79 (1.21)	0.624	0.93 (1.41)	0.91 (1.41)	0.760
	AA	1.46 (1.87)	1.57 (1.84)	0.043	1.66 (2.13)	1.75 (1.99)	0.349
	Dao	2.10 (1.56)	2.28 (1.60)	0.011	2.01 (1.70)	2.22 (1.86)	0.181
PWV (m/s)	Global	6.41 (1.50)	6.98 (1.72)	0.002	6.81 (1.46)	7.32 (1.84)	0.013
	AAo + AA	8.12 (3.54)	6.61 (3.15)	< 0.001	8.00 (3.58)	6.82 (2.625)	0.100
	Dao	5.99 (2.15)	7.20 (3.43)	0.005	5.75 (1.28)	6.99 (2.74)	< 0.001
Helicity	Aao	0.33 (0.02)	0.31 (0.03)	< 0.001	0.34 (0.02)	0.32 (0.02)	< 0.001
	AA	0.30 (0.03)	0.27 (0.03)	< 0.001	0.32 (0.03)	0.28 (0.04)	< 0.001
	Dao	0.28 (0.03)	0.29 (0.03)	0.010	0.30 (0.03)	0.30 (0.03)	0.009

*N* number of subjects, *AAo *ascending aorta, *AA* aortic arch, *DAo* descending aorta, *PWV* pulse wave velocity.

^a^Mean (± standard deviation).

^b^Wilcoxon signed rank test.

**Figure 3:**
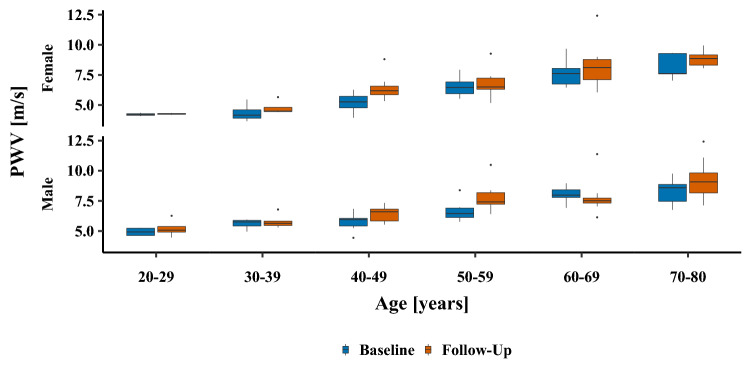
Progression of PWV after 6 years in the different decades. Blue box plots represent pulse wave velocities (PWV) in m/s at baseline, red box plots represent values six years later of the same study participants (lower and upper hinges correspond to the 25th and 75th percentiles). Please note that age on the x axis represents age of subjects at baseline. Values of females are given in the upper, those of males are given in the lower row.

Global PWV significantly increased in females and males (+ 0.57 ± 1.11 and + 0.51 ± 1.18 m/s, respectively) and similar results were found for regional PWV of the DAo but in a higher magnitude (+ 1.2 and + 1.25 m/s, respectively). The relative mean increase of PWV of the DAo was higher (+ 13%) compared to that of global PWV (+ 7.5%). We also calculated regional PWV of the AAo + AA. However, it revealed many outliers, obvious calculation errors and the decrease over 6 years was not plausible and in contrast to the results of global and regional PWV of the descending aorta. The annual progression of global PWV was 0.09 ± 0.17 m/s per year, assuming a linear progression over time. Changes of global PWV per decade in females and males are illustrated in Fig. 3. The increase of PWV was higher in older compared to younger subjects. In particular, subjects in the 40–49, 50–59, and 70–80 age groups (at baseline) showed a strong increase in PWV.

**Figure 4:**
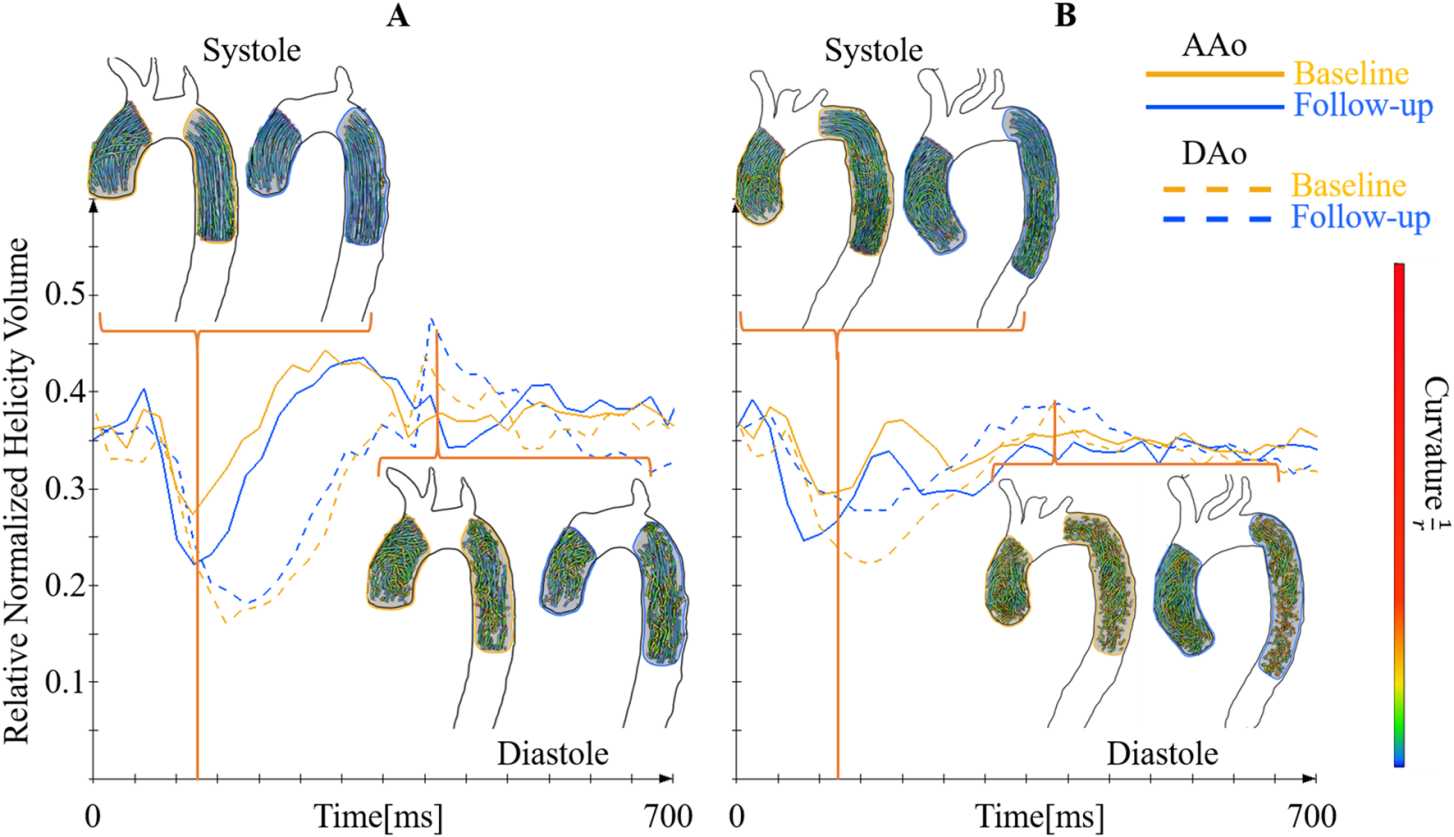
Changes in three-dimensional helicity over time. Examples for the development of the normalized thresholded relative helicity volume in the AAo and DAo segments are given in 27 year-old ((**A**) left diagram) and a 79 year-old subject ((**B**) right diagram). The yellow lines show the relative normalized helicity volume curves at baseline examination, the blue lines represent follow-up examination. The 3D visualizations show corresponding flow patterns as pathlines. The difference in the amount of helical flow in the ascending aorta (solid line) during systole is lower in the older subject. The difference in helicity between systole and diastole in the descending aorta increased in both subjects over time.

Finally, we observed that helicity decreased significantly in the AAo and AA while it increased in the DAo of both females and males (see Table 3). Differences in local normalized helicity volumes (LNHV) are displayed for each decade, for females and males both at baseline and follow-up in the supplemental Table 1. In addition, absolute values of helicity can be found in the supplemental Table 2. This table also includes temporal changes of helicity during the cardiac cycle, i.e. in systole (= systolic), early diastole (early diastolic) and in late diastole (diastolic). Finally, Fig. 4 illustrates changes of helicity during the cardiac cycle in a younger and in an older subject both a baseline and follow-up. The curves show that the LNHV portion decreased in the ascending aorta and decreased in the descending aorta in both follow-up examinations.

Multivariate regression analysis considering age, sex, hypertension (binominal) and PWV at baseline revealed that higher age at baseline was an independent predictor for increasing diameter of the aortic arch (p = 0.004). Male sex was an independent predictor for the enlargement of the diameter of the ascending aorta (p = 0.033). Hypertension at baseline was a predictor of decrease in atherosclerotic plaque thickness in the ascending aorta (p = 0.006). Higher baseline PWV was an independent predictor of a decreasing diameter of the AAo (p = 0.011) and AA (p = 0.001) and a decrease of helicity in the DAo (p = 0.013). None of the parameters above, including age, aortic diameter, and plaque thickness at baseline, independently predicted an increase in PWV.

### Measurement accuracy of PWV calculations based on 4D flow MRI

**Figure 5:**
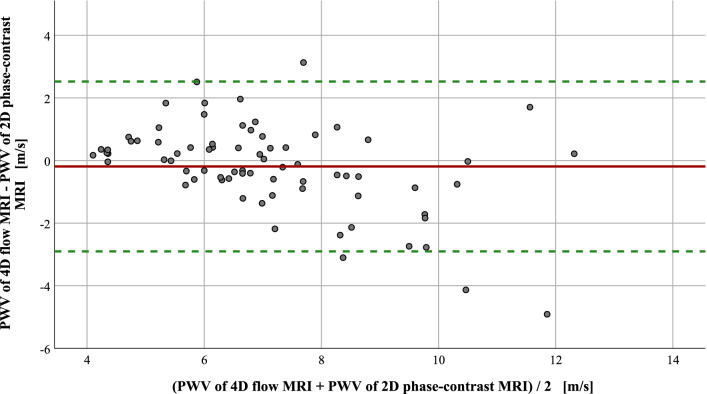
Comparison of PWV determined by 4D flow versus 2D phase-contrast MRI at follow-up. The Bland–Altman plot demonstrates that values of 2D phase-contrast MR angiography were minimally higher compared to values from 4D flow MRI (mean (solid line) = − 0.19; 1.96 × standard deviation (= 1.38) results in + 2.53 (upper limit, dashed line) and − 2.90 (lower limit, dashed line). This comparison of both MRI methods was possible in 74 patients.

Figure 5 demonstrates PWV values based on 4D flow MRI compared with 2D phase-contrast MRI. Mean difference was −0.19 m/s (1.96 × standard deviation = −2.90 to 2.53 m/s) indicating that values of 4D flow MRI were slightly lower compared to 2D phase-contrast MRI. The intraclass correlation coefficient for PWV determined by 4D flow MRI and 2D phase-contrast MRI was 0.76 indicating excellent agreement of both methods.

**Figure 6:**
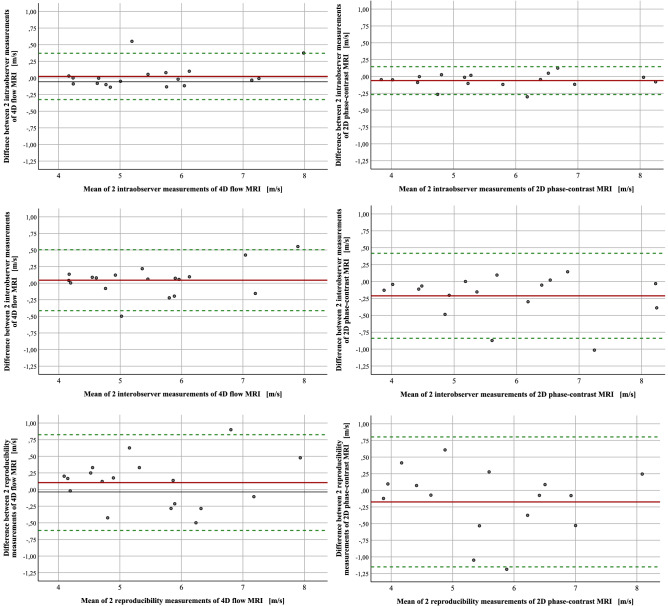
Measurement accuracy of PWV based on 4D flow and 2D phase-contrast MRI. Mean difference (solid line) ± 1.96 × standard deviation (dashed lines) is shown. Intra-observer agreement of 4D flow MRI (left column, n = 18) was 0.02 m/s [−0.32; 0.37], inter-observer agreement of 4D flow MRI was 0.04 m/s [−0.41; 0.5] and reproducibility of 4D flow MRI was 0.1 m/s [−0.62; 0.82]. Intra-observer agreement of 2D phase-contrast MRI (right column, n = 17) was −0.06 m/s [−0.27; 0.15], inter-observer agreement of 2D phase-contrast MRI was −0.21 m/s [−0.84; 0.42] and reproducibility of 2D phase-contrast MRI was −0.17 m/s [−1.15; 0.8].

Intra- and inter-observer agreement and reproducibility of PWV calculated on the basis of 4D flow MRI in 18 volunteers were high (difference 0.1 m/s and 1.96 × standard deviation was −0.62 to 0.82 m/s) (see Fig. 6). Interestingly, measurement accuracy was even higher in all these categories compared to 2D phase-contrast MRI although values were obtained with higher spatial and temporal resolution in MRI but at only three sites throughout the thoracic aorta. The intraclass correlation coefficient of scan-rescan reproducibility was 0.95 (4D flow MRI) and 0.93 (2D phase contrast MRI) for PWV calculation showing excellent reliability of both methods (see Fig. 6).

## Discussion

In this study, we followed 80 participants of a population study six years after baseline examination and measured changes of aortic diameter, wall thickness, global and regional pulse wave velocity and helicity. To our knowledge, this is the first study providing such detailed and age-specific longitudinal data acquired by high-resolution multi-parametric 3D MRI. The increase in global PWV by 0.54 m/s (ca. 8%) compared to the mean baseline PWV of 6.6 m/s six years earlier was the main and most reliable finding of our study. It represents a loss of aortic compliance due to aortic aging and/or progressive aortic atherosclerosis. In addition, changes of segmental helicity were robust and clear: helicity decreased in the ascending aorta and aortic arch over six years, which again is most probably due to a stiffening of the aorta and/or the increasing peripheral resistance with age. By contrast, helicity of the descending aorta increased which is not a contradiction. We demonstrated the increase of blood flow reversal at the inner curvature of the proximal DAo with age in the same population and the associated retrograde helical blood flow is most likely responsible for the observed increase of helicity in the DAo^26^. TTE was normal in almost all patients. Maximum velocity across the aortic valve increased significantly from 1.22 ± 0.21 to 1.34 ± 0.32 m/s but these values are normal for the aortic valve and do not indicate stenosis. Thus, we can state, that changes in PWV and helicity during follow-up are related neither to aortic valve stenosis, insufficiency nor to changes of left ventricular systolic function. We detected no enlargement of aortic diameters but a mild progression of aortic plaques in females. Finally, we were able to demonstrate the high measurement accuracy of 4D flow MRI for the calculation of PWV in additional 18 healthy volunteers. Measurement accuracy was even superior to PWV calculations based on 2D phase-contrast MRI although providing superior spatial and temporal resolution.

### Aortic diameter and aortic atheroma

Surprisingly, the diameter of the ascending aorta of women decreased significantly during six years. Most cardiovascular risk factors increased over time (see Table 1) and thus cannot explain this observation. Moreover, we did not intervene by a study-related medication or lifestyle modification. A measurement error is unlikely due to the large diameter of the ascending aorta of ca. 3 cm, the standardized fashion of measurement, and the high spatial resolution of 3D T1 weighted MRI of 1 mm^3^. Finally, mean left ventricular ejection fraction was the same at follow up and thus does not explain lower diameters of the ascending aorta at follow-up. Therefore, we believe that this observation has no biological correlate and is rather related to individual changes in our cohort and to the limited sample size.

As expected, plaque thickness increased during follow-up. It is plausible that changes were lowest in the ascending and highest in the descending aorta as shown by previous studies using transesophageal echocardiography, CT or MRI^13,27–29^. The inner curvature of the aorta is the predilection site of low wall shear stress. In addition, oscillating shear stress occurs in the proximal descending aorta due to diastolic blood flow reversal^26,30^ and both parameters are associated with the development and progression of local atherosclerosis^31^. The increase of plaque thickness was very similar to that in a previous study using 2D MRI in the mid descending aorta in a population study^10^. Overall, the progression was low which is due to the generally low incidence of cardiovascular risk factors in our participants.

### Increase in pulse wave velocity

Mean PWV of the thoracic aorta increased by 0.54 m/s (= 8.4%) during six years compared to the mean PWV of 6.6 m/s at baseline and this progression was found both in women and men and also for the regional PWV of the descending aorta in our cohort. Jarvis et al. quite recently analyzed aortic PWV of 99 adults aged 46 ± 15 (19–79) years in a transversal population study using 4D flow MRI and a similar spatial but lower temporal resolution (38.4–43.2 ms)^32^. They detected an increase of PWV by 1 m/s per decade and no differences in PWV between men and women which is very similar to the results in our longitudinal study. The MESA study^12^ investigated the development of PWV in 1160 participants of the population in six cities of the USA over 10 years. Mean age was 60 ± 9 years and thus higher than in our population. Accordingly, the increase in PWV was slightly higher, i.e. + 18% in 10 years or 10.8% in six years if a linear progression is assumed. Carotido-femoral tonometry is the most commonly used technique to assess PWV. It is easy to apply but also includes the compliance of the carotid artery, iliacal and femoral arteries and of the abdominal aorta^11^. The MESA study also measured PWV using 2D phase-contrast MRI at two single locations in the ascending and descending aorta perpendicular to the aortic lumen and at the level of the right pulmonary artery. Similarly, we focused on the thoracic aorta but used 4D flow instead of 2D phase-contrast MRI for longitudinal observation. Accordingly, we were also able to measure regional PWV. Spatial and temporal resolution in our study were < 2.5 mm^3^ and 20 ms, respectively. However, the short ascending aorta and aortic arch and the outlet of the supra-aortic arteries in the aortic arch obviously hampered calculation: PWV was higher in the ascending aorta and aortic arch compared to the DAo, which is not plausible due to the higher number of elastic fibers in the proximal aorta providing the Windkessel function. PWV of the DAo showed a more pronounced progression over time than global PWV but was only in part higher than global PWV although representing a muscular type artery. Soulat et al. comprehensively assessed PWV in 57 volunteers using carotido-femoral tonometry, 2D phase-contrast MRI and 4D flow MRI with a spatial and temporal resolution of ca. 2 mm^3^ and 34 ms, respectively^33^. Here, PWV of the ascending aorta was lowest and PWV of the descending aorta highest which is plausible and demonstrates the potential of 4D flow MRI for measuring regional PWV of the aorta. Our explanation for the differences between both studies is the disruptive effect of vessel outlets in the aortic arch and the limited length of the aortic segments in our study disturbing proper PWV calculation. Thus, resolution of 4D flow MRI and segment length should be as high as possible and the aortic arch should be excluded. The additional benefit of regional PWV of the thoracic aorta is currently unclear and needs to be established by further studies.

### Helicity of aortic blood flow

4D flow MRI allows to measure helical blood flow volume, index, length or other indicators to qualify helical flow. However, the physiological range and predictive value of these indicators is currently unknown^6^. The study by Ebel et al.^17^ and our longitudinal analysis now provide data of helical flow in larger groups of normal subjects that are ready for age-matched comparisons in patients. The segments defined for the AAo and DAo in our study were smaller than those defined by Garcia et al.^7^ allowing a comparison only for the aortic arch. The absolute volumes in our study at baseline (systole: 5.10 ± 2.03 ml, early diastole: 7.25 ± 2.55 ml and diastole 7.47 ± 2.38 ml) and follow-up (systole: 5.86 ± 2.60 ml, early diastole: 9.08 ± 3.82 ml and diastole: 10.23 ± 3.78 ml) correspond well to reported values of the control group^7^. The values for the AAo and DAo are lower than in this control group as expected because of the smaller segments (see supplemental Table 2). The voxel-based calculation of local normalize helicity (LNH) is dependent on spatial resolution and sensitive to velocity-encoding and preprocessing. This might limit the clinical applicability. However, for the given acquisition and preprocessing protocol we assume that the segment-based results allow a comparison with high reliability between baseline and follow-up in our population. To limit the influence of the segmentation variability in the aorta and to take anatomical difference between subjects into account, we analyzed relative LNH volumes.

### Measurement accuracy of PWV

Measurement accuracy of PWV in our healthy volunteers` study was high including a reproducibility with a difference of only 0.1 m/s as pointed out in Fig. 6. Moreover, we found excellent agreement of PWV calculation both using 4D flow and 2D phase-contrast MRI. This underlines that even considering measurement errors, our approach allows a very reliable monitoring and quantification of PWV progression over time. Notably, PWV values derived from 4D flow MRI were very similar to those calculated with the widely applied 2D phase-contrast MRI, which provides a twice as high spatial and temporal resolution.

### Predictors of the change of aortic diameter, wall thickness, PWV and helicity

In our population, male sex and increasing age were independent predictors for the progression of the diameter of the ascending aorta and aortic arch, respectively, which is plausible due to the progression of atherosclerosis. However, none of the parameters above and neither aortic diameter nor plaque thickness independently predicted a progression of PWV. A progression of diameter of the ascending aorta was reported in the systematic review of Tosello et al.^34^ and related to age, body size and especially hypertension. Higher baseline PWV was a weak but independent predictor for a decrease of helicity in the descending aorta. Accordingly, ageing or progressive atherosclerosis obviously lead to unfavorable effects in the thoracic aorta such as loss of compliance and helicity which is considered atheroprotective. In conclusion, higher age and male sex seem to be risk factors for the progression of aortic diameter and higher PWV for a loss of helicity in the descending aorta which may be one explanation for the higher incidence of complex atheroma at this location^19,26–29^.

### Limitations

Our study included 126 patients at baseline and 80 patients at follow up which is an acceptable drop-out rate in six years and when compared to similar studies^9^. The follow-up period of six years was too short to measure an increase of aortic diameter in this general population. Furthermore, we were not able to identify independent predictors for the progression of aortic diameters and atherosclerotic plaques. Investigation of larger cohorts with extended duration of follow-up, use of increased spatial resolution in MRI and automatic quantification of these parameters by dedicated software are therefore required. In addition, a reproduction of our findings in larger cohorts, at different study sites and using different software for off-line data analysis is necessary. The introduction of accelerated measurement of 4D flow MRI such as compressed sensing^35^ will save scan time and allow MR measurements with higher spatial and/or temporal resolution of 4D flow MRI resulting in further improvement of measurement accuracy of PWV and helicity.

## Conclusions

Multi-parametric 3D MRI is ideally suited for a comprehensive assessment of morphology and hemodynamics in the thoracic aorta and we present such MRI data on aortic aging in a population study. Aortic PWV and helicity determined by 4D flow MRI seemed to be more robust and sensitive for the determination of aortic aging and monitoring of the progression of atherosclerosis compared to aortic diameter and wall thickness. We believe that our age-related and long-term data are highly valuable and will serve as a reference to future comparisons with age-matched patients with diseases of the aortic valve or aorta. This will allow monitoring both the success of individual treatment and identifying a pathological progression of aortic pathologies.

## Supplementary Information

Supplementary Table 1.

Supplementary Table 2.

Supplementary Legends.

## Data Availability

The datasets generated during and/or analysed during the current study are available in the supplemental table. Further data is available from the corresponding author on reasonable request.

## Abbreviations

2D: Two-dimensional
3D: Three-dimensional
4D: Four-dimensional
AA: Aortic arch
AAo: Ascending aorta
BCA: Brachiocephalic artery
CCA: Common carotid artery
DAo: Descending aorta
GRAPPA: GeneRalized Autocalibrating Partial Parallel Acquisition
LNHV: Local normalized helicity volumes
MRI: Magnetic resonance imaging
PEAK: Parallel MRI with Extended and Averaged GRAPPA Kernels
PWV: Pulse wave velocity
TTE: Transthoracic echocardiography
TTF: Time-to-foot
XCor: Cross-correlation

## References

[1] Palombo C, Kozakova M (2016). Arterial stiffness, atherosclerosis and cardiovascular risk: Pathophysiologic mechanisms and emerging clinical indications. Vascul. Pharmacol..

[2] Sethi S, Rivera O, Oliveros R, Chilton R (2014). Aortic stiffness: Pathophysiology, clinical implications, and approach to treatment. Integr. Blood Press. Control.

[3] Smulyan H, Mookherjee S, Safar ME (2016). The two faces of hypertension: Role of aortic stiffness. J. Am. Soc. Hypertens..

[4] Townsend RR (2015). Recommendations for improving and standardizing vascular research on arterial stiffness: A scientific statement from the American Heart Association. Hypertension.

[5] Mancia G (2013). 2013 ESH/ESC guidelines for the management of arterial hypertension: The task force for the management of arterial hypertension of the European Society of Hypertension (ESH) and of the European Society of Cardiology (ESC). Eur. Heart J..

[6] Liu X, Sun A, Fan Y, Deng X (2015). Physiological significance of helical flow in the arterial system and its potential clinical applications. Ann. Biomed. Eng..

[7] Garcia J, Barker AJ, Markl M (2019). The role of imaging of flow patterns by 4D flow MRI in aortic stenosis. JACC Cardiovasc. Imaging.

[8] van Ooij P (2021). Fully quantitative mapping of abnormal aortic velocity and wall shear stress direction in patients with bicuspid aortic valves and repaired coarctation using 4D flow cardiovascular magnetic resonance. J. Cardiovasc. Magn. Reson..

[9] Bons LR (2020). Growth of the thoracic aorta in the smoking population: The Danish Lung Cancer Screening Trial. Int. J. Cardiol..

[10] Liu CY (2015). Evolution of aortic wall thickness and stiffness with atherosclerosis: Long-term follow up from the Multi-Ethnic Study of Atherosclerosis. Hypertension.

[11] Vlachopoulos C, Aznaouridis K, Stefanadis C (2010). Prediction of cardiovascular events and all-cause mortality with arterial stiffness: A systematic review and meta-analysis. J. Am. Coll. Cardiol..

[12] Ohyama Y (2016). Ten-year longitudinal change in aortic stiffness assessed by cardiac MRI in the second half of the human lifespan: The multi-ethnic study of atherosclerosis. Eur. Heart J. Cardiovasc. Imaging.

[13] Markl M (2010). Estimation of global aortic pulse wave velocity by flow-sensitive 4D MRI. Magn. Reason. Med..

[14] Markl M, Wallis W, Strecker C, Gladstone BP, Vach W, Harloff A (2012). Analysis of pulse wave velocity in the thoracic aorta by flow-sensitive four-dimensional MRI: Reproducibility and correlation with characteristics in patients with aortic atherosclerosis. J. Magn. Reason. Imaging.

[15] Wentland AL (2013). Aortic pulse wave velocity measurements with undersampled 4D flow-sensitive MRI: Comparison with 2D and algorithm determination. J. Magn. Reason. Imaging.

[16] Harloff A (2018). Determination of aortic stiffness using 4D flow cardiovascular magnetic resonance—A population-based study. J. Cardiovasc. Magn. Reson..

[17] Ebel S (2022). Quantitative normal values of helical flow, flow jets and wall shear stress of healthy volunteers in the ascending aorta. Eur. Radiol..

[18] Lang RM (2005). Recommendations for chamber quantification: A report from the American Society of Echocardiography’s guidelines and standards committee and the Chamber Quantification Writing Group, developed in conjunction with the European Association of Echocardiograph. J. Am. Soc. Echocardiogr..

[19] Harloff A (2012). 3D MRI provides improved visualization and detection of aortic arch plaques compared to transesophageal echocardiography. J. Magn. Reason. Imaging.

[20] Tunick PA, Kronzon I (2000). Atheromas of the thoracic aorta: Clinical and therapeutic update. J. Am. Coll. Cardiol..

[21] Markl M (2007). Time-resolved 3D MR velocity mapping at 3T: Improved navigator-gated assessment of vascular anatomy and blood flow. J. Magn. Reason. Imaging.

[22] Wehrum T, Kams M, Schroeder L, Drexl J, Hennemuth A, Harloff A (2014). Accelerated analysis of three-dimensional blood flow of the thoracic aorta in stroke patients. Int. J. Cardiovasc. Imaging.

[23] Drexl J, Mirzaee H, Harloff A, Hüllebrand M, Hennemuth A, Hahn HK (2013). A software tool for the computation of arterial pulse wave velocity from flow-sensitive 4D MRI data. Comput. Cardiol..

[24] Garcia J, Barker AJ, Collins JD, Carr JC, Markl M (2017). Volumetric quantification of absolute local normalized helicity in patients with bicuspid aortic valve and aortic dilatation. Magn. Reason. Med..

[25] R Core Team. *R: A Language and Environment for Statistical Computing*. https://www.R-project.org (R Foundation for Statistical Computing, 2021).

[26] Harloff A (2019). Retrograde aortic blood flow as a mechanism of stroke: MR evaluation of the prevalence in a population-based study. Eur. Radiol..

[27] Strecker C, Günther F, Harloff A (2020). Who should rather undergo transesophageal echocardiography to determine stroke etiology: Young or elderly stroke patients?. Front. Neurol..

[28] Chatzikonstantinou A (2012). CT angiography of the aorta is superior to transesophageal echocardiography for determining stroke subtypes in patients with cryptogenic ischemic stroke. Cerebrovasc. Dis..

[29] Katsanos AH (2014). Complex atheromatous plaques in the descending aorta and the risk of stroke: A systematic review and meta-analysis. Stroke.

[30] Markl M, Brendecke SM, Simon J, Barker AJ, Weiller C, Harloff A (2013). Co-registration of the distribution of wall shear stress and 140 complex plaques of the aorta. Magn. Reason. Imaging.

[31] Slager CJ (2005). The role of shear stress in the generation of rupture-prone vulnerable plaques. Nat. Clin. Pract. Cardiovasc. Med..

[32] Jarvis K (2022). Aortic pulse wave velocity evaluated by 4D flow MRI across the adult lifespan. J. Magn. Reason. Imaging.

[33] Soulat G (2020). Changes in segmental pulse wave velocity of the thoracic aorta with age and left ventricular remodeling. An MRI 4D flow study. J. Hypertens..

[34] Tosello F, Leone D, Laurent S, Veglio F, Milan A (2016). Out of proportion proximal aortic remodeling: A subclinical marker of early vascular ageing? A systematic review. Int. J. Cardiol..

[35] Pathrose A (2021). Highly accelerated aortic 4D flow MRI using compressed sensing: Performance at different acceleration factors in patients with aortic disease. Magn. Reason. Med..

