# Supplementary material for: Characterization of aortic aging using 3D multi-parametric MRI-long-term follow-up in a population study
Source: Sci Rep. 2023 Apr 18;13:6285. doi: 10.1038/s41598-023-33219-7 (PMC10111081; doi:10.1038/s41598-023-33219-7)
Supplement: Supplementary file 1 — Supplementary Table 1. [file 41598_2023_33219_MOESM1_ESM.docx]

| **Age at baseline**  **Number of subjects** | **20-29 years**  **6** | **30-39 years**  **10** | **40-49 years**  **14** | **50-59 years**  **19** | **60-69 years**  **18** | **70-80 years**  **13** |
| --- | --- | --- | --- | --- | --- | --- |
| **Baseline - females** |  |  |  |  |  |  |
| - Ascending aorta | 0.340±0.022 | 0.351±0.026 | 0.320±0.016 | 0.325±0.025 | 0.324±0.016 | 0.321±0.025 |
| - Aortic arch | 0.282±0.023 | 0.305±0.023 | 0.305±0.016 | 0.303±0.035 | 0.305±0.023 | 0.303±0.026 |
| - Descending aorta | 0.256±0.012 | 0.256±0.029 | 0.259±0.009 | 0.288±0.026 | 0.301±0.015 | 0.306±0.025 |
| **Baseline - males** |  |  |  |  |  |  |
| - Ascending aorta | 0.353±0.030 | 0.322±0.031 | 0.338±0.020 | 0.325±0.021 | 0.328±0.016 | 0.341±0.009 |
| - Aortic arch | 0.326±0.047 | 0.298±0.024 | 0.313±0.030 | 0.303±0.045 | 0.324±0.020 | 0.327±0.027 |
| - Descending aorta | 0.275±0.025 | 0.269±0.018 | 0.286±0.021 | 0.288±0.020 | 0.313±0.016 | 0.312±0.022 |
| **Follow-up - females** |  |  |  |  |  |  |
| - Ascending aorta | 0.320±0.050 | 0.321±0.022 | 0.306±0.020 | 0.306±0.032 | 0.311±0.026 | 0.305±0.020 |
| - Aortic arch | 0.216±0.013 | 0.264±0.026 | 0.272±0.023 | 0.263±0.025 | 0.288±0.023 | 0.276±0.017 |
| - Descending aorta | 0.259±0.007 | 0.258±0.027 | 0.280±0.020 | 0.294±0.022 | 0.308±0.020 | 0.310±0.026 |
| **Follow-up - males** |  |  |  |  |  |  |
| - Ascending aorta | 0.327±0.036 | 0.299±0.025 | 0.322±0.015 | 0.322±0.019 | 0.311±0.024 | 0.318±0.017 |
| - Aortic arch | 0.260±0.033 | 0.244±0.023 | 0.278±0.036 | 0.278±0.039 | 0.300±0.035 | 0.292±0.035 |
| - Descending aorta | 0.277±0.023 | 0.279±0.012 | 0.300±0.029 | 0.315±0.022 | 0.317±0.045 | 0.303±0.039 |

**Supplemental table 1.** Mean values ± standard deviation of local normalized helicity volumes (LNHV) in the three aortic segments are given in females and males both at baseline and follow-up.
