# Supplementary material for: Characterization of aortic aging using 3D multi-parametric MRI-long-term follow-up in a population study
Source: Sci Rep. 2023 Apr 18;13:6285. doi: 10.1038/s41598-023-33219-7 (PMC10111081; doi:10.1038/s41598-023-33219-7)
Supplement: Supplementary file 3 — Supplementary Legends. [file 41598_2023_33219_MOESM3_ESM.docx]

**Supplemental tables legends**

**Supplemental table 1.** Mean absolute values ± standard deviation of normalized values of helicity in the three aortic segments are given in females and males both at baseline and follow-up.

**Supplemental table 2.** Absolute and normalized values of helicity are given in the three aortic segments in females and males both at baseline and follow-up. *AAo* = ascending aorta, *AA* = aortic arch, *DAo* = descending aorta. *Volume* means absolute volume of helicity, *LNHVolume* means relative volume of helicity normalized to the individual volume of the aortic segment. *Systolic* represents helicity in systole, *early diastolic* incorporates helicity in early diastole and *diastolic* helicity stands for helicity in late diastole of the cardiac cycle.
